# Supplementary material for: Micro-size plankton abundance and assemblages in the western North Pacific Subtropical Gyre under microscopic observation
Source: PLoS One. 2021 Apr 26;16(4):e0250604. doi: 10.1371/journal.pone.0250604 (PMC8075241; doi:10.1371/journal.pone.0250604)
Supplement: Attachment — Submitted filename: PONE_D_20_37689_Review.docx [file pone.0250604.s001.docx]

PONE-D-20-37689 Review

Title: Micro-size plankton abundance and assemblies in the western North Pacific Subtropical Gyre under microscopic observation

Authors: Taketoshi Kodama, Tsuyoshi Watanabe, Yukiko Taniuchi, Akira Kuwata, and Daisuke Hasegawa

Summary: Authors called for a re-evaluation of the micro-size plankton community in the western NPSG to understand the hetero/homogeneity of their community structure. They collected plankton samples over 3 years (2016-2018) in the euphotic zone (<200 m) and analyzed via microscopy. It is commendable the amount of work that was conducted for this manuscript, using traditional methodology such as microscopy. The work is interesting in that the authors utilized several modeling approaches to elucidate drivers of specific microplankton populations in the NPSG. This work is comprehensive and thorough, despite its claiming that it was a “side project” during expeditions that had an alternative main focus. I believe that it will be a strong contribution to the field, with several key edits that I recommend below. Also, most of the manuscript is written in intelligible fashion and in standard English, except for a few choices of words (e.g. heterogenous) that are not correct, and also the use of parentheses to convey patterns of the opposite trend is confusing. In regards to these examples, the manuscript must be revised to be made more clear.

Major comments:

-This paper provides an overview of microplankton diversity and community structure in the western NPSG through microscopy. The method used is not necessarily novel, and I would have liked to see a combination or groundtruthing of more cutting edge techniques (e.g. DNA metabarcoding) with classic methodologies such as microscopy. I know that is not possible, so perhaps there could be a comparison with studies done with DNA in the area or similar areas to provide this context.

-Production measurements of microplankton would also provide greater understanding of the microplankton contribution and importance to ecosystem functioning. There are several references to this in the Discussion, but there must be historical measurements of carbon fixation in the area? This would also lean into the fisheries discussion that will set this study in a broader context.

-Many of the figures need more explanation in the Results. A few lines to explain the GAMs (Fig. 6-7), in terms of the basics (additive effect, the scales for each panel and how they differ), would be beneficial for the reader.

-I think that a Table showing the depths of the SCMs during each cruise (which is specified in L 226-228) would be more clear, or label the depths in the figures. It is also confusing that the SCM was 94 +/- 10 m in KY1704, but it is plotted beneath 100 m in the plots. I see you need to bin the samples this way because the samples weren’t always taken at the same depth, but it is a bit misleading to see a “depth profile” without the same range between the values on the y-axis. You could also try depth-integrating all the parameters to a specific light bin – 0-100m, 100 m to SCM, SCM to 200 m to break up the parameters around the Chlorophyll a profile. Where is your 1% surface light level? You could alternatively break it up according to light, but this may not be important for the heterotrophic folks – though they would likely be driven by their photosynthetic prey.

-Perhaps the SCM values in the Table could be combined with how many samples were taken at each depth and each cruise? You state that 127 total samples were taken – what is the breakdown (of the box plots in Figs. 3 and 4)? Also, are the environmental parameter sample count the same as the plankton sample count (in Fig. 2)?

Minor Comments:

Title: do they mean “assemblages” versus “assemblies”?

Abstract:

why is a re-evaluation necessary? Maybe one line expanding on what you describe in Introduction:

L31: Peridiniales?

L40: heterogeneous?

L40-43: major conclusions are lacking – the focus of the study is about reevaluating the microplankton, and the major conclusion from the abstract indicate nitrogen fixation is a large contributor.

-Introduction:

L47-49: Instead of using the parentheses, break up the pico vs micro comparison into another sentence.

L60: heterogeneous

Methods:

L96: were these measurements taken on an expedition that had a primary aim to investigate Japanese eel larvae? What does it mean that “other observations were not always organized well in terms of space and time”?

L115: fluorescence

L128: assemblages?

L168-L177: are there satellite altimetry showing sea level anomaly in this study region?

Results:

L234: nitracline is not the depth where there is 1 uM of nitrate concentration, itʻs the region of rapid change in nitrate concentration relative to depth. So for your cruises it looks like between 100-150 m.

Figure 1: the y-axis should be plotted on a linear scale, relative to the SCM. What are the SCM-High, SCM, and SCM-Low depths for each cruise? – applies to all the figures. Or have a table that tells the readers what the SCM depths are for each cruise?

L239-240: why do you think fluorometer values were overestimating the chl a concentration in the deeper layers?

Figure 4: It’s difficult to compare abundances, do they go from highest to less from left to right?

Figure 5: (A) Dinophyceae spelled with a y

L393 and 396 both start with “On the other hand…”

Figure 8b: Dinophyceae speeled with a y

L428: TEM should be TEMP

Discussion:

L466-468: Is this contribution of >10 um chl a concentration integrated from all depths sampled, or per depth? I would think that chl a concentrations and contributions would differ in varying light conditions (e.g. photoadaptation) so this must be evaluated accordingly with respect to light fields.

L515 and other places: When there is the polar opposite condition in parentheses, itʻs confusing. Please clarify.

L554-556, 562, 564, 574: heterogenous means “of foreign origin” and not the same as heterogeneous, which is the word I think you are trying to use.

L573: heterogenetic is not a word, I believe.

L579 to end: This ending sentence about eDNA for migratory fish comes out of nowhere – a few lines linking the study of microphytoplankton to migratory fish (like you do in Intro) in the Discussion would be beneficial.

Acknowledgments:

Any funding to acknowledge?
