# Supplementary material for: Micro-size plankton abundance and assemblages in the western North Pacific Subtropical Gyre under microscopic observation
Source: PLoS One. 2021 Apr 26;16(4):e0250604. doi: 10.1371/journal.pone.0250604 (PMC8075241; doi:10.1371/journal.pone.0250604)
Supplement: Attachment — Submitted filename: Response_letter_PONE-D-20-37689-R2.docx [file pone.0250604.s003.docx]

**Response to Reviewer**

In this version, the authors have addressed all of the comments of the reviewers to satisfaction. I have made few, mostly grammatical suggestions here:

**We thank the reviewer’s comments. I revised the manuscript as suggested.**

L5 typo in the short title

**We revised from “Micro-sizes” to “Micro-size” (L5).**

L18-20 Edit first sentence to: “While primary productivity in the oligotrophic North Pacific Subtropical Gyre (NPSG) is changing, the micro-size plankton community has not been evaluated in the last 3 decades, prompting a re-evaluation.”

**We revised as suggested (L18-20).**

L23 Don’t need s in community structures.

**We revised from “community structures” to “community structure” (L22) as well as L556.**

L24 “The assemblages were consistent with those identified 4 decades previously.” Also 4 decades? Thought the first sentence indicated 3 decades.

**We revised from “3 decades” to “4 decades” of the first sentence (L19).**

L25 Dinophyceae were “the most numerically abundant, followed by Cryptophyceae and Bacillariophyceae (diatoms)”.

**We revised as suggested (L25).**

L42 Therefore, nitrogen fixation “may contribute” to the heterogeneity …

**We revised as suggested (L41).**

L49-50 In other words, energy fixed by primary production is more efficiently transferred to higher trophic organisms in micro-plankton dominated waters.

**We revised as suggested (L48-50).**

L61 is instead of as

L62 phytoplankton are “the dominant primary producers”;

**We revised “is the dominant primary producers” (L61-62).**

L64 abundant but is it significant? What % of cell counts or Chl a?

**We added “(> 10^8^ cells m-2) in the surface mixed layer” with a new reference Scharek et al. 1999 DSRI (L64).**

L69 western NPSG is oligotrophic (no ‘the’)

**We revised as suggested (L69).**

L70 migratory fish?

**We revised as suggested (L70).**

L88-89 To understand the trophic structure and heterogeneity of this community.

**We revised as suggested (L88-89).**

L93 three cruises on the R/V …

**We revised as suggested (L93).**

L98-99 therefore, the observations for collections of seawater were limited to night-time collections.

**We revised as suggested (L98-99).**

Table 1 is very informative and much improved, thank you!

**We also thank the comments.**

L121 – collected using … (no “by”)

**We revised as suggested (L121).**

L244 Nitrate concentration was over 1 uM of in water “where” the temperature was below

**We revised as suggested (L245).**

L250 …layers as indicated “by” Falokowski …

**We revised as suggested (L250).**

L472 …primary production in the wester NPSG depicted slight increase.

**We revised as suggested (L472).**

L480 The metabarcoding results were consistent “with” our microscopic observation, with the exception of Dinophyceae which comprised a larger proportion of the community in metabarcoding results. This is likely due to Dinophyceae having largernuclear genome sizes compared to other microplankton.

**We revised as suggested (L481-483).**

L484-490 This observation suggests that while microscopic observation is a time consuming technique, it is still a necessary tool to understand the micro…..; furthermore, this indicates that the metabarcoding technique should best be coupled with more traditional methods such as microscopic observations.

**We revised as suggested (L485-489).**
